# Low-temperature reaction dynamics of paramagnetic species in the gas phase

**DOI:** 10.1039/d1cc06394d

**Published:** 2022-02-21

**Authors:** Lok Yiu Wu, Chloé Miossec, Brianna R. Heazlewood

**Affiliations:** The Oliver Lodge, Department of Physics, University of Liverpool Oxford Street Liverpool L69 7ZE UK b.r.heazlewood@liverpool.ac.uk; Physical and Theoretical Chemistry Laboratory, Department of Chemistry, University of Oxford South Parks Road Oxford OX1 3QZ UK

## Abstract

Radicals are abundant in a range of important gas-phase environments. They are prevalent in the atmosphere, in interstellar space, and in combustion processes. As such, understanding how radicals react is essential for the development of accurate models of the complex chemistry occurring in these gas-phase environments. By controlling the properties of the colliding reactants, we can also gain insights into how radical reactions occur on a fundamental level. Recent years have seen remarkable advances in the breadth of experimental methods successfully applied to the study of reaction dynamics involving paramagnetic species—from improvements to the well-known crossed molecular beams approach to newer techniques involving magnetically guided and decelerated beams. Coupled with ever-improving theoretical methods, quantum features are being observed and interesting insights into reaction dynamics are being uncovered in an increasingly diverse range of systems. In this highlight article, we explore some of the exciting recent developments in the study of chemical dynamics involving paramagnetic species. We focus on low-energy reactive collisions involving neutral radical species, where the reaction parameters are controlled. We conclude by identifying some of the limitations of current methods and exploring possible new directions for the field.

## Abstract 2

By carefully controlling the reactants and precisely probing the products, we can gain insights into how radical reactions occur on a fundamental level.

## Graphical abstract

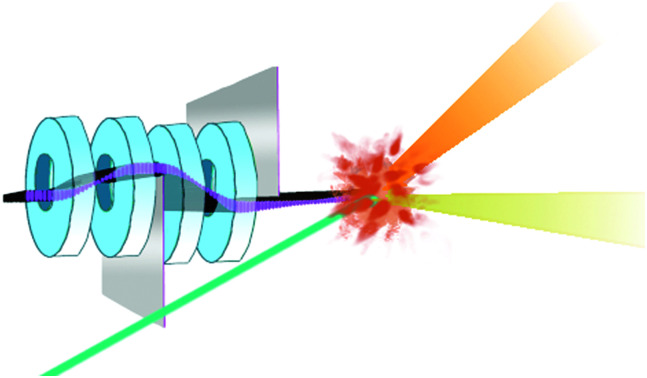

## 1 Introduction

Atoms and molecules with one or more unpaired electrons in the outermost orbital are often referred to as ‘paramagnetic’ or ‘radical’ species. Paramagnetic species are important in a range of gas-phase processes: they are involved in reactions occurring in the atmosphere, in plasmas, in combustion systems, in flames, and in the interstellar medium (ISM). Indeed, radicals are directly responsible for much of the chemistry occurring in these complex gas-phase environments. For example, OH has been described as the “vacuum cleaner” and the “detergent” of the troposphere, such is the ubiquity of hydroxyl radicals in atmospheric oxidation pathways.^1,2^ The release of free Cl radicals upon the breakdown of chlorofluorocarbons by ultraviolet radiation in the stratosphere—resulting in the well-documented destruction of ozone—is another example of the important gas-phase chemistry driven by radical species.^3^ Many of the >240 molecular species unambiguously identified in interstellar regions are paramagnetic,^4^ including the first molecule detected in the ISM, methylidyne (CH).^5,6^ Molecular oxygen is typically paramagnetic, due to the triplet nature of the ground state, and is critical for combustion processes. Many other radical species (such as OH, HO_2_, C(^3^P), and CH) are frequently formed as combustion by-products. It is apparent that, in order to accurately model the chemistry occurring in these (and other) complex and diverse gas-phase environments, we need to first understand the reactivity of key radical species.

Beyond the direct applications to real-world gas-phase environments, studying the chemical reactions of radical species is also of fundamental interest. As a result of having a magnetic dipole moment, arising from the spin of the unpaired electron(s), paramagnetic species in selected quantum states can be manipulated by external magnetic fields. This property (amongst others) makes it possible to exert a significant amount of control over radical reactants. As discussed in the subsequent sections of this article, a variety of experimental methods have been developed to examine how radical species react—enabling the reaction conditions to be precisely controlled and the reaction products to be detected with high sensitivity. These approaches have paved the way for detailed chemical dynamics investigations; reaction mechanisms, product branching ratios and even product state distributions are now well understood for a number of radical reaction systems. Coupled with these advances in experimental techniques, theoretical work has also seen significant advances in recent years. Owing to the transient nature of most reaction intermediates, experimental measurements typically involve the detection of final reaction products. It is therefore useful to combine experimental findings with detailed calculations (such as electronic structure calculations and the construction of potential energy surfaces upon which trajectories can be propagated) to confirm the validity of proposed reaction mechanisms and provide a complete picture of the reaction process, from reactants to products.

In some reaction systems, theory work has challenged preliminary experimental findings—resulting in additional measurements and calculations, and typically yielding a deeper understanding of the reaction dynamics. Such was the case in the reaction of Cl(^2^P) with *para*-H_2_. Due to the unpaired electron in the Cl p shell, orbital and spin angular momenta interactions give rise to two spin–orbit states, Cl(^2^P_3/2_) and Cl(^2^P_1/2_). These two spin–orbit states exhibit different reactivity, owing to differences in the orientation of the unpaired p electron with respect to the H_2_ reactant. Early experimental work indicated that spin–orbit excited Cl(^2^P_1/2_) reacted more readily with H_2_ than ground state Cl(^2^P_3/2_)—in contrast to theoretical predictions based on the Born–Oppenheimer (BO) approximation.^7,8^ A feature common to many of the reaction systems discussed here is the presence of more than one low-lying potential energy surface (PES), and the need to consider possible non-BO behaviour (such as non-adiabatic effects) to account for the chemical dynamics observed. It took almost a decade for subsequent experiments, coupled with quantum scattering calculations on high-level PESs, to confirm that the BO predictions were valid and that ground state Cl reacts much more readily than spin–orbit excited Cl.^9^ A theme seen throughout this highlight article is the complexity present in the chemical dynamics of what initially appear to be ‘simple’ radical reactions. The specific reasons behind the complexity, and the effects on reaction mechanisms, are examined below for a number of reaction systems.

In this article, we discuss and review the reaction dynamics of selected neutral paramagnetic species. The focus is on experimental studies of reactions at low collision energies, and on methods where the properties of one (or both) of the reactants are explicitly controlled. As will be detailed in the following sections, by controlling the properties of the reaction it becomes possible to unravel the contributions made by each of the variable parameters. In this way, the importance of (for example) collision energy or internal energy in a given system can be established—in many cases, enabling the reaction mechanism to be unambiguously ascertained and revealing details that were hidden in room-temperature studies. The desire to exercise control over the reaction parameters has seen significant experimental progress made in recent years. For a handful of systems, the recording of state-to-state measurements (*i.e.*, combining the preparation of state-selected reactants with state-selective product detection) is now a reality. Instead of compiling a list of the different radical reaction systems that have been studied to date, we have attempted to showcase some of the most interesting and recent results. We discuss how these studies have advanced our understanding of radical reactivity and conclude by identifying some possible future directions for the field.

### 1.1 Background theory

Arrhenius first proposed an equation for calculating the temperature dependence of reaction rate coefficients as long ago as 1889, *k* = *A*e^−*E*_a_/*RT*^ (with *k* the rate coefficient, *A* the pre-exponential factor, *E*_a_ the activation energy, *R* the gas constant and *T* the temperature).^10^ The pre-exponential term in the Arrhenius equation (*A*) accounts for the frequency of collisions between the reactants; the e^−*E*_a_/*RT*^ term accounts for the fraction of collisions that have sufficient energy to surmount the activation energy barrier. Over the past several decades, a range of models have been developed in an attempt to account for the dynamics underlying the Arrhenius expression. One such model, known as simple collision theory, sets out that the frequency with which two reactants collide is dependent on their relative speed and the collision cross section. The collision cross section, *σ*, can be estimated by treating the reactants as hard spheres, *σ* = π*d*^2^, where their collision diameter, *d*, is the sum of the radii of the colliding reactants. While simple collision theory is straightforward to calculate, it is not terribly accurate in predicting the probability that a reaction will occur. Aside from approximating (often complex) molecules as hard spheres, the orientation of the reactants and the nature of the collision—as described by the impact parameter, *b*—are typically important considerations.

All collisions must conserve energy, linear momentum and angular momentum. However, not all collisions are reactive. In many cases, only kinetic energy or internal energy is exchanged between colliding reactants—giving rise to elastic and inelastic scattering, respectively. Reactive collisions occur when the products of the collision are chemically distinct from the reactants. Collision cross sections can be calculated to account for the different probabilities associated with each of these processes. One can even calculate state-to-state reaction cross sections, *σ*_*if*_, to describe the probability of a reactant in a selected quantum state, *i* = (*j*, *v*), forming a state-selected product, *f* = (*j*′, *v*′), at a given collision energy (where *j* and *v* represent the rotational and vibrational quantum numbers, respectively). In some cases, it is possible to control the orientation of radical reactants (for example, by using magnetic fields) and to examine the effect this has on the outcome of a collision. Certain detection methods can also ascertain how products are scattered following a reaction. In the following paragraph, some frequently encountered types of cross sections and collisions are described. While these concepts will be familiar to many readers, definitions are provided for those less familiar with the terminology.

The term ‘differential cross section’ refers to the direction in which a product is scattered with respect to the velocity vector of one of the reactants. The differential cross section, d*σ*/d*Ω*, is calculated as the fraction of particles that are scattered in the solid angle *Ω*. An ‘integral cross section’ can then be established by integrating over the scattering angle (*θ*) of the products and the azimuthal angle (*ϕ*) at which the reactants collide. Measuring the differential cross section of a reaction can sensitively probe the reaction dynamics. In many cases, as detailed below, the combination of detailed experimental measurements with high-level theoretical calculations of differential cross sections can reveal the mechanism responsible for a given reaction process. For example, direct reaction pathways that proceed *via* head-on collisions are typically characterised by low impact parameters, a small reaction cross section (*i.e.*, a low likelihood of reaction), and backward-scattered products. In contrast, processes that involve long-lived reaction complexes—where the lifetime of the intermediate species is larger than the rotational period of the complex—do not typically exhibit a strong correlation between impact parameter and scattering direction.^11,12^

**Fig. 1:**
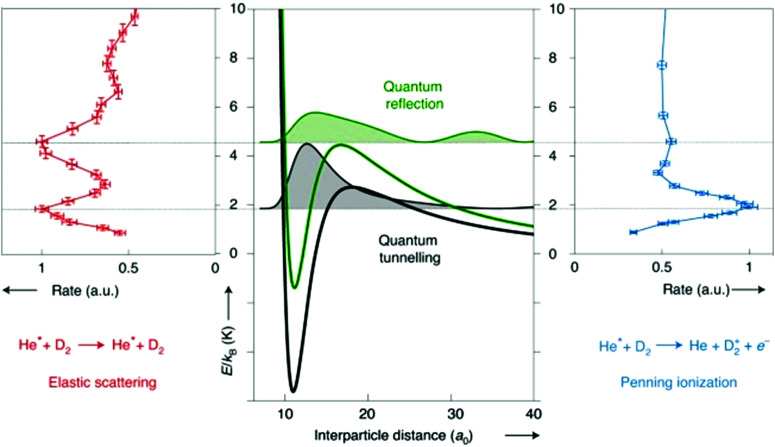
Schematic illustration of the different types of resonances present in the Penning ionisation and elastic scattering pathways following collisions between metastable He and D_2_. Note that metastable He, denoted He* in the figure, is a triplet radical species. At collision energies that coincide with the location of partial waves (with the squared resonance wavefunctions shown shaded in black and green on the central panel), peaks are seen in the rate coefficient plots. Adapted from Paliwal *et al.*, copyright 2021, under exclusive licence to Springer Nature Limited.^13^

As more control is being exerted over the reactants, with ever-lower collision energies, more and more quantum effects are being observed in radical reaction dynamics. Many of these quantum features arise due to the presence of resonances, and can be seen as features in plots of the cross section or reaction rate coefficient *versus* collision energy (at low energies; see Fig. 1). Resonance states are typically not fully-bound states, but rather correspond to quasi-bound states of the collision complex. Different types of resonances can be present in a given system, depending on the precise location of the resonance. For example, shape and orbiting resonances can occur on single potential energy surfaces that feature a (submerged or energetic) barrier along the reaction pathway. Shape resonances are often associated with processes that result in tunnelling through the barrier (or quantum reflection above the barrier), usually arising from intermolecular vibrations within the complex. Orbiting resonances are typically associated with short-lived collision complexes and delocalised wavefunctions, and refer to resonances that are localised on or above a barrier (by analogy with the classical picture of ‘orbiting’, as the colliding species can be thought of as orbiting around one another). Feshbach resonances occur when there is coupling to a bound state of a nearby potential energy surface—for example, as can be seen in the formation of a highly vibrationally excited dimer upon the association of two ultracold atoms under certain conditions—and are associated with highly localised resonance wavefunctions.^13,14^ As can be seen in the examples highlighted below, the ability to resolve these quantum features—both experimentally and in theoretical work—is becoming achievable for an increasing number of systems.

This highlight article identifies some of the key advances made in the study of reaction dynamics involving paramagnetic reactants over the past decade or so. While the article is structured around the different experimental techniques that are typically employed, it is important to also acknowledge the critical role that theory work has played in developing our understanding of reaction dynamics. Electronic structure calculations have been in widespread use for many decades now, with a range of *ab initio* methods employed to calculate accurate PESs for a multitude of reaction systems. Over the past several decades, advances in both experimental and theoretical methods have seen new insights into fundamental chemical reactivity. Some of the early experimental achievements, that underpin the more recent developments, are set out in the following subsection. For a comprehensive discussion of the theoretical characterisation of bimolecular reaction dynamics in the gas phase, readers are directed to several excellent perspective articles (and references cited therein); only a few key points are noted here.^15–19^ In order to accurately describe the reaction dynamics occurring in low-energy and state-selected collisions, the interaction potential must precisely account for both long-range and short-range intermolecular forces. This can be difficult to achieve for radical reaction systems, due to the open-shell character of the paramagnetic reactant(s). While significant advances have been made in the development of electronic structure theory methods for open-shell species, some challenges remain. For example, calculations for even three-atom systems can become computationally intractable in the presence of external fields (such as magnetic fields).^16,20^ In spite of these challenges, recent years have seen some remarkable advances in the size and complexity of systems successfully described using state-of-the-art theoretical methods—with several investigations explicitly discussed in this highlight article.

### 1.2 Early experimental studies

The study of chemical reaction dynamics in the gas phase was transformed by the introduction of the crossed molecular beam method. “The dynamics of chemical reactions—a fascinating new field of research” was the title of the press release for the 1986 Nobel Prize in Chemistry, awarded to Herschbach, Lee, and Polanyi.^21^ In a review article published the following year, Lee eloquently set out the importance of crossed molecular beams in advancing our understanding of elementary chemical reaction dynamics.^22^ In particular, the use of skimmed supersonic beams with narrow velocity distribution, narrow angular spread and narrow internal state distribution has been critical in unveiling previously hidden details. Alongside the development of crossed molecular beam methods, the widespread adoption of laser-based techniques has enabled reactants to be prepared in specific quantum states and products to be state-selectively detected.^23^ Of particular relevance to this highlight article, lasers can also be employed to form beams of gas-phase radicals through the photodissociation of closed-shell molecular precursor species. Other methods frequently used for the generation of gas-phase radical reactants include electron beam irradiation methods and electrical discharges.^24–27^ It is also important to note the foundations laid by electron paramagnetic resonance (EPR) studies conducted in the 1960–1970s, with EPR measurements identifying the properties of many gas-phase radicals—including information on the Zeeman effect.^28–30^

Armed with methods for preparing radical reactants and probing product formation, the study of radical reaction dynamics began in earnest. Many book chapters and review articles on gas-phase chemical dynamics feature reactions involving radical species.^14–19,22,31–36^ And yet, many decades after the first experimental studies of radical reaction dynamics, there are still unanswered questions about how paramagnetic species react—both in terms of the general behaviour exhibited, and when considering specific reaction systems. For example, the F + H_2_ reaction system has been extensively and actively studied, both experimentally and theoretically, for more than 50 years.^37–40^ What may initially appear to be a simple exothermic abstraction reaction, F + H_2_ → HF + H, is complicated by the presence of multiple potential energy surfaces, owing to the open-shell nature of the radical reactant. The BO approximation is known to break down in regions where two or more PESs become degenerate (or are very close in energy), requiring the consideration of non-adiabatic (BO-forbidden) processes to fully account for the experimental observations.^41^ A combination of high-level crossed molecular beam studies and full quantum mechanical calculations involving multiple PESs (including spin–orbit and Coriolis coupling) was needed to explain the chemical dynamics at play. The evolution in our understanding of the F + H_2_ reaction (with some of the most recent findings discussed in the following subsection) beautifully showcases how seemingly simple reaction processes—involving as few as three atoms—can turn out to be surprisingly complex.

New methods, improvements to existing methods, and new combinations of techniques, have seen incredible advances in recent years. The reaction dynamics of a range of radical reaction systems can now be studied in detail—with excellent control over the reaction parameters and sensitive detection of the reaction products. The following sections highlight some of the methods frequently adopted for the experimental investigation of radical reaction dynamics. A number of reaction systems are discussed in detail, accompanied by more general comments about our current understanding of radical reactivity in the gas phase and the limitations of current methods.

## 2 Radical reaction dynamics

### 2.1 Crossed molecular beams

As mentioned in the preceding section, the crossed molecular beam method has been central to the study of bimolecular reaction dynamics. In a crossed molecular beam apparatus, two (typically supersonic) beams intersect in a vacuum chamber at an angle *α*, with the resulting collision energy determined by the equation1
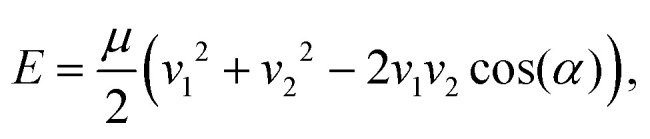
where *v*_1_ and *v*_2_ is the velocity of reactants 1 and 2 (respectively), with *μ* the reduced mass of the reactants. The majority of early work on crossed molecular beams involved crossing angles of 90°, giving rise to relatively high collision energies. More recent efforts have looked at reducing the angle between the crossed beams and varying the beam velocities, in order to control and tune the energy at which the reactants collide. Crossed molecular beam reaction dynamics have been extensively reviewed in the past, including in several recent articles;^15,32,42^ readers are directed to these resources for a more in-depth discussion of the method and the reactions studied in this way.

**Fig. 2:**
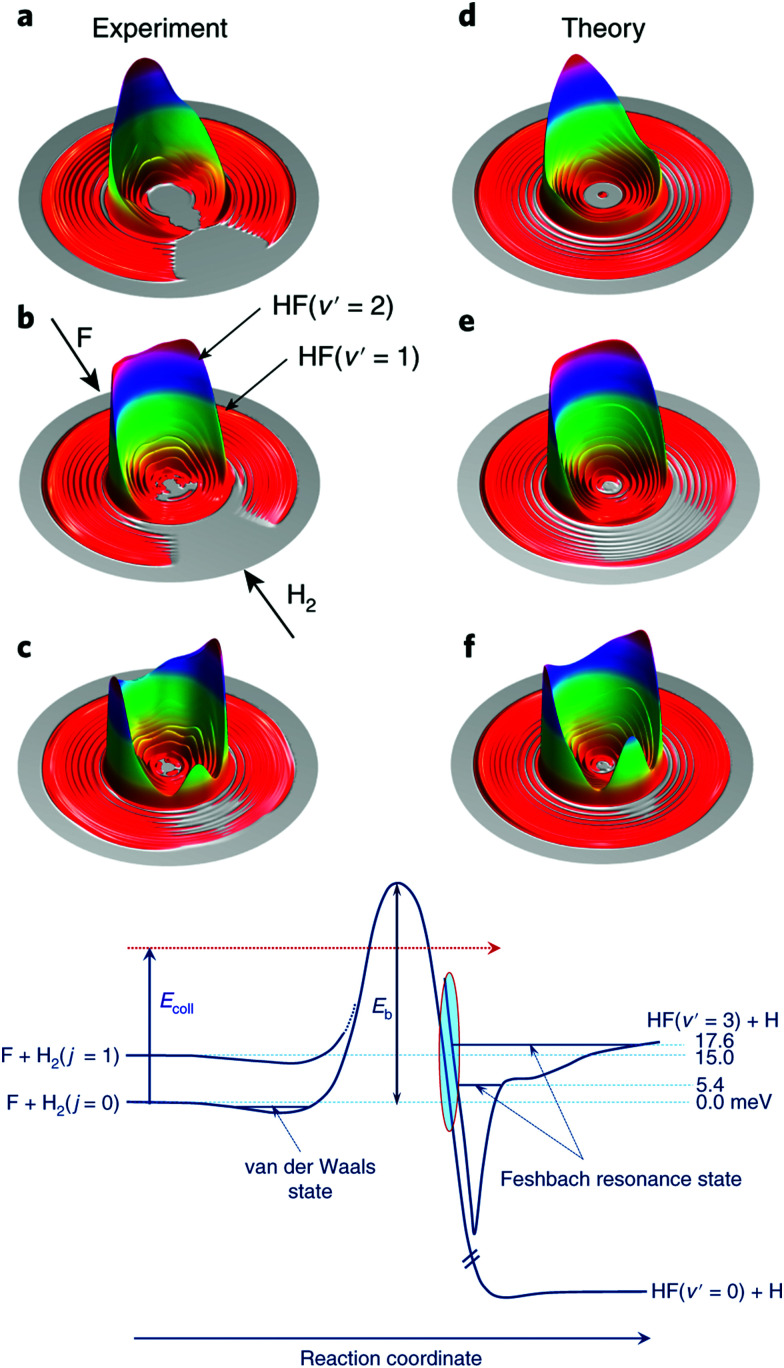
Experimental (a–c) and theoretical (d–f) differential cross sections are presented as three-dimensional contour plots of the product velocity, at three collision energies (top: 1.56 meV, middle: 6.93 meV, bottom row: 9.97 meV). Below the differential cross section plots, the features of the reaction pathways for the reaction of H_2_(*j* = 0) and (*j* = 1) with F are shown schematically. Feschbach resonance states can be seen in the exit channel (after the reaction barrier). Reproduced from Yang *et al.*, copyright 2019, under exclusive licence to Springer Nature Limited.^44^

As touched on in the preceding section, the reaction between F and H_2_ has been a benchmark system for the study of radical reaction dynamics. The crossed molecular beam method has been critical to deepening our understanding of the F + H_2_ → HF + H reaction process. A seminal 2007 study found that, at low collision energies, spin–orbit excited F(^2^P_1/2_) reacted with D_2_ approximately 1.6 times faster than ground state F(^2^P_3/2_).^41^ This is despite the fact that the reaction of F(^2^P_1/2_) is forbidden within the BO framework, while that of F(^2^P_3/2_) is allowed. Breakdown of the BO approximation was most pronounced at low collision energies, due to the presence of a small reaction barrier along the BO-allowed pathway. At low collision energies, the reaction was found to proceed almost entirely as a result of D-atom tunnelling through the energetic barrier.^41^ More recent work has examined the role of resonances in dictating the outcome of reactive collisions between F and H_2_.^43,44^ The reaction is the only known source of HF in interstellar clouds and is known to proceed under low-temperature (10–100 K) conditions, in spite of the presence of a significant activation energy barrier.^45^ By altering the angle (down to 26°) between the F and *para*-H_2_ reactant beams, and by modifying their relative velocities, the collision energy was able to be tuned from 1.21–35 meV. The preference for quantum mechanical tunnelling at low energies was attributed to the presence of a post-barrier resonance state (see Fig. 2).^44^ Through isotopic substitution, even further details on the reaction dynamics have subsequently been elucidated. For example, vibrationally excited D_2_ reactants exhibited different relative reactivity with the two spin–orbit states of F (compared to ground state D_2_). Differential cross section measurements (at collision energies spanning 13.9–113.6 meV) revealed the DF products to be backward scattered at the lowest collision energies, with the formation of side-scattered products preferred as the collision energy was increased, and forward scattering becoming dominant in the most vibrationally excited products, DF(*v*′ = 5).^46^

Ultra-high resolution velocity map imaging techniques, combined with near-threshold ionisation of the product D atoms, has enabled partial-wave resonances to be resolved in the reaction of F with HD.^47^ A “horseshoe-shaped” pattern was identified in the (rotational-state-resolved) differential cross sections in forward-scattered HF products. In a bid to account for this observation, full spin–orbit considerations were included in the quantum dynamics calculations. Excellent agreement was found between the experimental and theoretical results when all angular momentum couplings—electron spin, electron orbital motion, angular momenta of the nuclei—were explicitly accounted for. When these spin–orbit effects were neglected, the horseshoe feature was absent from the theoretical differential cross section plots.

The horseshoe pattern was found to arise from quantum interference, with spin–orbit partial-wave splitting occurring at low energies (where partial-wave resonances were already known to play an important role). The total angular momentum, **j**, is established by taking the vector sum of the spin angular momentum, **s**, and the orbital angular momentum, **l**. Here, **s** and **l** also couple to the nuclear orbital angular momentum, **L**, giving rise to the additional spin–orbit splitting observed.^48^ Even after decades of attention, the combination of cutting-edge experimental investigations and state-of-the-art theory work has unveiled previously hidden features in the benchmark F + H_2_ reaction system.^47^

The identification of a range of other quantum effects—including quantum interference and geometric phase effects in H + HD → H_2_ + D, and Feschbach resonances in the F + H_2_O → HF + OH reaction—further highlights the importance of precise experimental measurements and high-level theory work in deepening our understanding of radical reaction dynamics.^49–51^ For example, quantum features observed in integral cross section measurements of the S(^1^D_2_) + H_2_ → SH + H insertion reaction, studied at low collision energies using crossed molecular beams with 22.5° ≤ *α* ≤ 62.5°, were in good agreement with theoretical predictions.^52^ However, when one of the H atoms was replaced by D (*i.e.*, in the S + HD reaction), the integral cross sections recorded at low collision energies were not immediately able to be described by theoretical predictions. It was proposed that accurate PESs and the inclusion of non-adiabatic effects were necessary to describe the low-energy features observed experimentally in the S + HD system.^53^

As the preceding two paragraphs clearly demonstrate, high-quality PESs are critical for the accurate calculation of radical reaction dynamics. Theory methods have progressed steadily over the past several decades, with high-quality *ab initio* PESs now able to be calculated for nine-atom systems.^54^ For example, the reaction dynamics of the Cl(^2^P_3/2_) + C_2_H_6_ system were able to be accurately established from quasi-classical trajectory calculations for the first time in 2020.^54^ All previous theoretical studies had been unable to account for the features observed in experimental measurements. It is challenging to accurately represent this system theoretically due to the relatively high number of atoms (and electrons) present, and the open-shell nature of the radical reactant. The calculation of a high-quality PES was achieved through the use of advanced electronic structure methods that describe electron correlation and consider the coupling of spin and orbital angular momenta.^54^ The zero-point-energy-corrected hydrogen abstraction reaction coordinate was found to be exothermic, featuring an adiabatically submerged barrier and yielding HCl + C_2_H_5_ products. (Interestingly, the pathway was found to be endothermic, with a positive reaction barrier, if zero-point energy was not included.) Product rotational state distributions and scattering angle distributions established from quasi-classical trajectory calculations were found to reproduce the features seen in experimental measurements,^55,56^ thereby resolving the previous (long-standing) disagreement between experimental and theoretical work on this system.^54^

While there have been a large number of radical reaction systems examined by the crossing of two supersonic beams (as identified above and in ref. 57–59, in addition to many other systems identified within recent review articles^15,32,42,60^), some exciting new work has seen the use of a Zeeman decelerator in a crossed beam set-up. Experiments have thus far been focused on inelastic scattering, with the exceptional energy resolution of the NO beam enabling diffraction oscillation to be observed (in NO + Ne collisions) and product-pair correlations identified (in NO + O_2_ collisions)—see subsection 2.4 for more details.^61^ The ability to very precisely control the quantum state and velocity of the paramagnetic reactant is an exciting prospect for future reaction studies. Stark decelerators and electrostatic hexapole guides have also been successfully utilised in the manipulation of radicals that also possess an electric dipole moment (such as OH and NO), with a number of inelastic scattering studies reported.^62–66^ A magnetic hexapole guide has also been adopted for crossed beam inelastic scattering studies involving paramagnetic N_2_(A^3^Σ_u_^+^) and NO(X^2^Π). Through the application of an additional magnetic field to align the metastable N_2_ reactants after they exited the hexapole, the effect of stereodynamics on the probability of energy transfer was explored. The external magnetic field, and the subsequent alignment induced in the metastable N_2_ reactants, had a significant effect on the probability of energy transfer in collisions with NO. This observation was explained by the enhanced overlap between the N_2_(
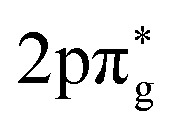
) and NO(6σ) molecular orbitals in certain configurations.^67^ While these examples involve inelastic scattering, there are exciting prospects for the use of external magnetic fields to control the properties of reactive radical collisions. Ongoing work into the study of collisions using decelerated beams is discussed in subsection 2.4 below.

### 2.2 Merged beams

A central theme in this highlight article is the ability to control the properties of radical reactants, in order to learn about their reaction dynamics. As was seen in the discussion of crossed molecular beam studies, low collision energies (alongside high-resolution detection methods) have been critical for the observation of resonances and quantum effects. The energy with which two beams collide is set out in eqn (1) above. Collision energy is minimised when the velocities of the two reactant beams are equal (*v*_1_ = *v*_2_) and the angle of intersection is zero (as cos(0) = 1). Instead of crossing two beams at a small angle, an alternative approach has seen two reactant beams merged—such that *α* = 0. The merging of two reactant beams (and controlling the velocities of the beams to be almost equal) has been achieved by the deflection of a paramagnetic beam onto a beam containing non-paramagnetic species, thereby achieving collision energies below 1 K.^68,69^ This approach to merging two supersonic beams has been very successfully applied to the study of Penning ionisation (and competing association) processes, and has already been examined in several review articles.^14,33,70–72^ For example, by carefully controlling and adjusting the velocity of the beams, collision energies below 10 mK were achieved in the merging of a metastable He beam with a beam of Ar or H_2_—enabling orbiting resonances to be observed in the resulting Penning ionisation reactions.^68^ Subsequent studies on merged H_2_ and He(^3^P_2_) beams identified a rotational state dependence in the rate of chemi-ionisation, with (rotationally excited) *ortho*-H_2_ reacting faster than *para*-H_2_ due to the presence of stronger long-range attractive forces.^73^ We direct readers to the review articles for an in-depth discussion of the technique and an overview of the systems studied to date.^14,33,70–72^ Here, we have chosen to focus our analysis on the most recent merged beam studies.

The role of stereodynamics has been explored in the merging of metastable Ne(^3^P_2_) with beams of rare gas atoms, N_2_, or CO, yielding Penning and associative ionisation products. The paramagnetic Ne species can be oriented (*i.e.*, the electronic orbital angular momentum of the atom can be controlled, which consequently orients the 2p orbital—as seen in the Cl + H_2_ study discussed in the introduction) by the application of a weak magnetic field at the end of the magnetic multipole guide. Rotating the direction of the weak external magnetic field has the effect of rotating the orientation of the Ne(^3^P_2_) reactants—enabling steric effects to be directly probed. No general trend was observed in the resulting stereodynamics; while some systems (such as reactions involving N_2_ and CO) displayed a dependence on the orientation of the Ne(^3^P_2_), this could only be accounted for by considering the properties of each individual system.^74–77^ The competition between Penning ionisation and associative ionisation in merged beams of Ar with metastable Ne(^3^P_2_) or He(^3^P_2_) has also been measured (in the absence of any magnetic orientation fields). The relative strength of long-range interactions in the different systems was found to be responsible for the branching ratios and reaction rate coefficients: Ne(^3^P_2_) has a stronger ion-induced dipole interaction with Ar than He(^3^P_2_), giving rise to an increased preference for Penning ionisation in the Ne(^3^P_2_) + Ar system.^78^

As has already been noted in this section, merged beam methods have facilitated the direct observation of a number of quantum effects, with a near-merged beam set-up (*α* = 4.5°) recently identifying evidence of Feshbach resonances as a result of coincidence measurements of different reaction products.^79^ An exceptional amount of detail about the state-to-state dynamics of a Penning ionisation reaction was uncovered by examining the correlation between the energy of an ejected Penning electron and the associated Penning ion product. The coincidence part of the measurement was crucial, as this enabled the ion-electron pairs to be correlated to a selected collision event. In this way, the Penning ionisation reaction between Ar and metastable He was studied—with both the Ar^+^ ionic product and the ejected electron detected. The additional details provided by the coincidence measurements enabled the initial and final quantum states to be “mapped” and a scattering resonance observed, with the detection of spin–orbit excited HeAr^+^ Feshbach molecules.^79^ The combination of a merged beam apparatus with a sensitive velocity map imaging set-up has enabled quantum resonances to be resolved in the (elastic) differential cross sections for collisions between metastable He and normal D_2_. The low collision energies and near-threshold photoionisation of the scattered He(^3^P_2_) products allowed diffraction oscillations to be observed, in addition to resonances causing a preference for backward scattering (and enhanced scattering rates) at selected collision energies. By combining the experimental measurements with scattering wavefunction calculations, both tunnelling and above-barrier resonances were able to be identified (see Fig. 1).^13^

A different approach to merging two reactant beams has been adopted to study the reactions of H and D with small molecular ions over a large energy range. The apparatus is comprised of two beamlines, with the neutral and ionic reactants prepared and manipulated separately before the beams are merged. In the neutral beamline, a duoplasmatron source produces anionic precursors (for example, D^−^ is formed from D_2_ by the duoplasmatron source, for the eventual formation of a neutral D beam). The anions are filtered and deflected, before the electron is photodetached. The beam of cations is also produced by a duoplasmatron source (to generate, for example, H_2_D^+^ from a mixture of H_2_ and D_2_ gas), with this beam also filtered and deflected prior to being combined with the neutral beam. This unique merged beam set-up has enabled, for example, rate coefficients for isotopic exchange in the collisions of D atoms merged with beams of H_3_^+^, H_2_D^+^ or D_2_H^+^ to be recorded over a large range of collision energies (3 meV–10 eV).^80–82^ The measured reaction rate coefficients were found to be consistent with the number of available deuteration sites in the different isotopic exchange systems studied.^80^ Interestingly, the experimentally derived rate coefficients exceeded those predicted by quasi-classical trajectory calculations and ring polymer molecular dynamics methods (with the calculations performed on a full-dimensional PES)—at all temperatures considered, and for all three reactions.^83^ Again, these findings highlight the importance of detailed experimental measurements over a range of conditions, even for processes as simple as H/D isotopic exchange.

Finally, while technically not involving a ‘merged’ beam, reactive collisions between radicals and closed-shell species in a single supersonic expansion have been examined. H or D radicals (formed by an electric discharge) were co-expanded with phenol and toluene, with the products detected spectroscopically. Exclusively *ortho* hydroxy- and methyl-cyclohexadienyl radical products were found following H- or D-addition reactions. This was attributed to the approximately 5 kJ mol^−1^ lower entrance channel barrier for the formation of *ortho* isomers when compared to *meta* or *para* isomers (and even lower when compared to the barrier to formation of *ipso* products).^84^

### 2.3 Flow tube methods

A flow-based technique known as CRESU (which stands for Cinétique de Réaction en Ecoulement Supersonique Uniforme [reaction kinetics in uniform supersonic flow]) was developed in the 1980s to perform rate coefficient measurements for ion–molecule (and subsequently also radical–neutral) reactions at low temperatures.^85,86^ At the heart of the CRESU technique is the Laval nozzle, an asymmetric hourglass-shaped nozzle consisting of convergent and divergent components, through which a gas expands isentropically and adiabatically from a reservoir. Laval nozzles produce a collimated, effectively wall-less flow of gas, reaching temperatures as low as 5.8 K.^52^ The beam has a well-defined and uniform pressure, temperature, and density for several tens of centimetres (in most systems).^85^ By switching Laval nozzles and buffer gases, a series of beams with different properties can be produced, allowing reactions to be investigated under different temperature regimes. Thanks to the development of pulsed CRESU methods, which have significantly lower pumping requirements, a number of CRESU set-ups now exist and a plethora of astronomically relevant radical–neutral reactions have been studied using CRESU over the past several decades. As the technique has been extensively covered by several previous review articles,^87–89^ this section will aim to highlight a handful of the radical reaction systems studied, focusing on recent advances and noting some potential future applications of the method.

The reaction of CN radicals with benzene (and its derivatives) are of interest due to the detection of benzonitrile in the ISM.^90^ The reactions of species such as benzene are expected to be important in the formation of polycyclic aromatic hydrocarbons (PAHs) in the ISM. Reactions that yield small cyclic organic molecules, such as benzonitrile, are postulated to serve as a ‘proxy’ for benzene—providing details about how benzene reacts in the ISM, and giving an indication of how much benzene might be present in different interstellar regions. (Benzene itself is very challenging to detect directly in the ISM, as it possesses no electric dipole moment.) In two independent studies,^91,92^ no temperature dependence of the rate coefficient was found in the reaction between CN and benzene from 15 K to 295 K. A barrierless entrance channel was identified, which gives rise to the formation of an addition complex. The reaction is efficient at all astrochemically relevant temperatures, with benzonitrile the dominant product formed. A related reaction system involving CN and toluene also displays a consistently fast rate coefficient over the temperature range 15–294 K, albeit with some discrepancy in the value of the rate coefficient (owing to experimental challenges in the preparation of reactants).^91,93^ The study of these (and related) radical reaction processes are improving our understanding of the chemistry occurring in the ISM, with the rate coefficients and product branching ratios of the CN + C_6_H_6_ system now included in the KInetic Database for Astrochemistry (KIDA).

The detection of products has long been a challenge in CRESU experiments, with laser-induced fluorescence (LIF) methods widely employed to monitor the rate of reactant consumption. In the S(^1^D_2_) + H_2_ → SH + H reaction, using LIF to monitor the decay of S(^1^D_2_) enabled the rate coefficient to be calculated, but additional crossed-beam experiments were necessary to obtain the integral cross sections for the reaction (as discussed in subsection 2.1).^52,94^ Product formation has been successfully monitored in some systems, providing more information about the reaction dynamics. In the reaction between C(^3^P) and CH_3_CN, both the rate of C(^3^P) decay and the formation of H(^2^S) products were (separately) detected.^95^ This allowed temperature-dependent product branching ratios to be determined—paving the way for possible future measurements of the internal energy distribution in products. The combination of CRESU methods with a chirped-pulse Fourier-transform microwave spectrometer (CP-FTMW) and a continuous-wave cavity ring down spectrometer have further extended the applicability of CRESU to the study of CN radical reaction dynamics.^96–99^

There have been significant advances in low-temperature studies of reactions involving radicals and oxygenated volatile organic compounds or complex organic molecules. Systems that have received particular attention include reactions such as OH + CH_3_OH, OH + CH_3_CHO, OH + CH_3_C(O)CH_3_, and CH + H_2_CO.^100–111^ While many of these CRESU studies have focused on the measurement of rate coefficients, in several cases interesting reaction dynamics were also observed. For example, several OH reaction pathways that involved tunnelling through the abstraction activation barrier were found to yield rate coefficients independent of pressure. In pathways where the rate coefficient did vary with pressure, the presence of a reaction intermediate was identified—with changes in pressure altering the extent of collisional stabilisation (and therefore the lifetime) of the reaction complex. The prevalence of OH in the atmosphere, and the need to account for the abundance and reactivity of OH in the ISM, has seen these reaction systems included in a number of databases and models of atmospheric and interstellar chemistry.

Another important astrophysical radical species is NH, which was first detected in the interstellar medium in 1991.^112^ Collisions between NH and H_2_, the most abundant molecule in the universe,^113^ have been studied both experimentally and theoretically. While the reaction is endothermic—with a negligible rate coefficient under interstellar conditions—understanding the inelastic collision dynamics is important for modelling the abundance of NH in the ISM and interpreting astronomical NH spectra. Potential energy surfaces have been calculated for the NH–H_2_ system,^114^ with the global minimum corresponding to a van der Waals complex with a linear configuration, with the N end of NH located beside the H_2_ moiety (in agreement with an earlier study).^115^ Excitation cross sections (with total energies up to 500 cm^−1^) were also calculated for NH with *ortho*- and *para*-H_2_. The calculations identified a preference for maintaining the orientation of the electron spin—with transitions that conserved electron spin exhibiting larger cross sections. These findings are consistent with other studies on the NH molecule;^116,117^ calculations at higher collision energies, in addition to further experimental work, are anticipated in the coming years.

The above paragraphs have considered the reactions of radicals with non-paramagnetic species. While studying radical–radical reactions is often technically challenging, the last few decades have seen a number of these systems successfully examined using CRESU methods. In 2005, the reaction between two radical species—ground-state OH radicals and O atoms—was measured and rate coefficients reported for temperatures spanning 39 to 142 K.^118^ O atoms were able to be generated in large excess by pulsed laser photolysis (using a VUV co-photolysis method), and the decay of OH signal was observed by LIF. Even at temperatures as low as 39 K, no significant change in the rate coefficient was found; the process remained fast at low temperatures. This is a feature typical of radical–radical reactions, as such systems are often barrierless and hence reactions can be fast at low temperatures. In the following decade, rate coefficients for the reaction of N atoms with NO (48–211 K),^119^ OH (56–296 K),^120^ CN (56–296 K),^121^ and CH (56–167 K)^122^ were reported. The combined findings have implications on N_2_ formation in the ISM, as reactions with OH and CH (and subsequent reactions with their products, NO and CN, respectively) represent two competing pathways for N atoms—in contrast to predictions included in KIDA at the time.^123–125^

In addition to CRESU, a number of other flow-based methods have been applied to the study of radical reaction dynamics. In particular, the selected ion flow tube (SIFT) technique has been widely used for the study of ion–radical reactions. Ions are first produced in the source (by methods such as electron impact) and are subsequently mass-selected by a quadrupole mass filter. The ions are then collisionally thermalised by helium buffer gas, following which radical reactants (produced by methods such as pyrolysis or microwave discharge) are injected into the flow tube. Selected products (or the consumption of reactants) can then be detected at the end of the flow tube, with ion signals typically measured. Due to experimental challenges such as condensation on the walls of the tube, most of the studies have been conducted at room temperature—although recent developments have seen variable-temperature set-ups introduced.^126^ Owing to the significant number of reactions studied using SIFT over the past several decades, and the typical focus on reaction kinetics (instead of dynamics), we have chosen to only highlight a few recent studies in the following paragraphs.

In a flowing afterglow SIFT experiment, several reactions of the diradical *ortho*-benzyne (*o*-C_6_H_4_) were examined at room temperature. The reaction channel *o*-C_6_H_4_ + OH^−^ → C_6_H_3_^−^ + H_2_O was found to proceed an order of magnitude slower than expected; exothermic proton abstraction pathways are usually barrierless processes that proceed rapidly to products.^127^ A competing associative electron detachment reaction pathway, where the neutral addition product is formed alongside the release of an electron, was proposed—accounting for the slower-than-expected proton abstraction process. The SIFT technique has been combined with an electrospray ionisation source to investigate the reactions of deprotonated nucleobases with atomic radicals at room temperature.^128^ Interesting differences were observed in the relative reactivity of H, N, and O with the deprotonated nucleobases: N was unreactive; reactions with O yielded several competing product channels (associative electron detachment, addition of O accompanied by loss of H, and formation of the OCN^−^ anion); and reaction with H proceeded rapidly by associative electron detachment. A number of studies involving the reactions of small cations with radicals including N, O, C_2_H_5_, H_2_C_2_F_3_, C_2_F_3_, and C_2_F_5_ have been undertaken using flow-based methods.^129,130^ While these studies again primarily focused on establishing rate coefficients, some interesting trends in reactivity were identified. For example, the probability of long-range charge transfer was found to be lower in several ion–radical reaction systems than in the corresponding reactions of ions with closed-shell counterparts. Despite this, charge transfer was still found to be the dominant process in systems where it was exothermic.

While falling outside the scope of this highlight article, it is also worth noting that a large number of atmospherically important radical reaction systems have been studied in flow cells at (or near) room temperature. For example, reactions between peroxy radicals and carbonyl oxides (also known as Criegee intermediates), which can lead to the formation of secondary organic aerosols in the troposphere, have recently been studied using cavity ring-down spectroscopy combined with laser flash photolysis in a flow cell.^131^ Readers are directed to a recent review article discussing the chemistry of Criegee intermediates for further details on these atmospherically important reaction pathways.^132^ Flow-based methods have provided a vast amount of experimental data on radical reactions over a range of temperatures—with many of the reaction properties incorporated into databases developed for astronomical and atmospheric modelling. An inherent limitation common to all flow-based methods, however, is that the reactions need to be relatively fast, as the measurement time is limited by the finite length of the tube. This has seen the development of complementary methods, with some of these (for example, the trap-based methods described in subsection 2.5) set out below.

### 2.4 Deceleration techniques

Crossed molecular beam and flow-based methods are very broadly applicable techniques for the study of bimolecular reactions. Neither of these approaches are specific to radical reactants—although, as has been set out above, both of these methods have been very successfully applied to the study of radical reaction dynamics in a number of different systems. There are several more targeted approaches now available that take advantage of the intrinsic properties of radicals (such as paramagnetism) to produce quantum state- and velocity-selected beams of paramagnetic species. This is the basis for manipulation techniques that use external field effects—such as Zeeman deceleration or magnetic guiding (using magnetic fields), or Stark deceleration (using electric fields)—to control the properties of radical reactants. In the presence of an external magnetic field, the energy levels of paramagnetic species are split. States that are raised in energy are termed low-field-seeking (LFS) states, while those that are lowered in energy are termed high-field-seeking (HFS) states.

Zeeman deceleration was first successfully demonstrated in 2007, with the production of low-velocity beams of metastable Ne and H through the use of time-varying, spatially inhomogeneous magnetic fields.^133,134^ In a conventional Zeeman decelerator, the atomic or molecular beam passes through a series of solenoid coils and the switching sequence (*i.e.*, the times at which currents are switched on and off for each coil) is timed such that paramagnetic species in LFS states experience a potential gradient. If the magnetic field (induced by the application of current to a coil) is switched off at an appropriate time, a small amount of kinetic energy can be permanently removed, causing the target species to slow down. By varying the switching sequence, one can decelerate the target radicals to a range of final velocities, whilst maintaining state selectivity in the beam. The Stark deceleration technique is the electric equivalent of Zeeman deceleration, using electric fields in place of magnetic fields. It was demonstrated five years earlier and has several technical advantages (as it is easier to switch high voltages rapidly than high currents), but is limited to species possessing an electric dipole moment.^135^ A few species that are paramagnetic and possess an electric dipole—such as OH and NO—have been successfully manipulated by both Stark and Zeeman decelerators.

There are well-acknowledged challenges associated with generating a sufficient number of state- and velocity-selected radicals for reaction studies.^136^ This is primarily because only a small subset of species within the incoming beam are successfully decelerated—comprising radicals in an appropriate LFS state, with a velocity and position that place them within the phase-stable ‘bunch’. The typical density of successfully decelerated beams is on the order of 10^7^ particles cm^−3^.^135,137^ While a number of crossed-beam experiments have been undertaken with Stark-decelerated radicals, these studies have focused on inelastic scattering processes.^16,138–142^ (While not involving a Stark decelerator, the Stark effect has been exploited in the study of reactive collisions between OH and Br, with a hexapole used to state-select the OH radical reactants. A negative collision energy dependence was found for the collision cross section, implying that there is no energetic barrier along the reaction pathway.^143^) Collision studies involving Zeeman-decelerated beams are a very recent advancement in the field and, to date, only inelastic collisions have been studied.^61^

**Fig. 3:**
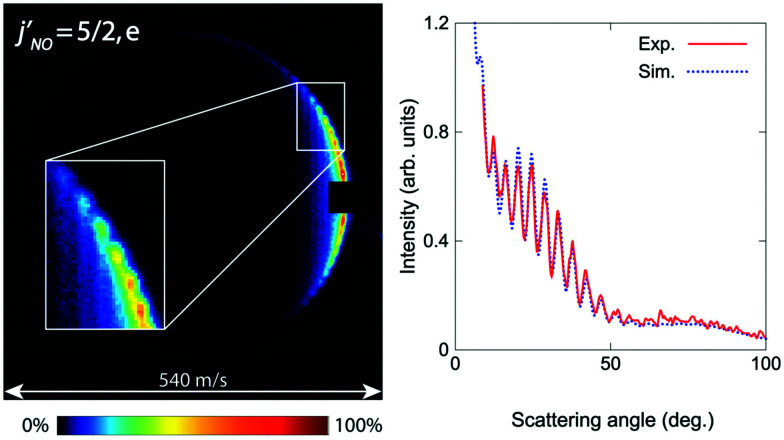
Velocity map scattering image recorded following inelastic collisions between NO(X^2^Π_3/2_, *j* = 3/2) and Ne. Oscillatory patterns due to matter wave diffraction can be observed. As can be seen in the right hand side plot, excellent agreement was found between the experimental and simulated results. Adapted with permission from Plomp *et al.*, copyright American Institute of Physics, 2020.^61^

The first crossed beam experiment using a Zeeman-decelerated beam was reported in 2020, probing inelastic scattering between Zeeman-decelerated NO(X^2^Π_3/2_, *j* = 3/2) and Ne or O_2_.^61^ Using a 2.2 m long Zeeman decelerator with alternating solenoids and hexapoles,^144^ the transverse and longitudinal focusing effects were effectively decoupled, producing a beam with variable longitudinal velocities and narrow transverse velocities. Inelastic collisions between NO and Ne yielded some interesting results, with the diffraction of matter waves causing oscillatory patterns to emerge in the state-to-state differential cross sections (see Fig. 3). This effect was particularly pronounced for collisions that caused a small change in the rotational angular momentum of NO.^141^ In contrast, strong product-pair correlations—where the excitation of one collision partner to a selected final state is associated with the formation of the other collision partner in a specific final state—were identified following collisions between NO and O_2_. The inelastic channels for rotational excitation were attributed to short-range head-on collisions, whereas long-range glancing collisions were more closely associated with elastic scattering.^145^ More recently, angular scattering distributions for inelastic collisions between Zeeman-decelerated C(^3^P_1_) and He atoms have been reported using a crossed-beam set-up.^146^ Oscillatory patterns were once again observed due to the diffraction of matter waves, with the angular scattering distributions in close agreement with simulations obtained from quantum-mechanical close-coupling calculations.

While reactions involving Zeeman-decelerated beams of radicals are yet to be successfully observed, a number of initiatives have succeeded in improving the output of Zeeman decelerators. With ever-improving detection sensitivity and the optimisation of Zeeman deceleration methods—in addition to innovative combinations of experimental techniques—the study of reactions with Zeeman-decelerated radicals is now becoming feasible.^34,71,147–149^ To this end, as discussed in the following sub-section, Zeeman (and Stark) decelerators have been successfully combined with a range of traps—facilitating the confinement of radical species for an extended period of time (up to 10s of seconds). Travelling-wave (or moving magnetic trap) Zeeman decelerators, where paramagnetic species are confined in three dimensions and progressively decelerated as they travel along a series of spatially overlapped quadrupole traps, are particularly suited to being combined with traps (due to their improved transverse stability compared to conventional Zeeman decelerators).^150,151^ Other recent developments in the deceleration of paramagnetic species includes the Zeeman-Sisyphus deceleration of a molecular species, CaOH, to velocities below 15 m s^−1^.^152^ Zeeman–Sisyphus deceleration works by passing paramagnetic species through a spatially-varying magnetic field produced by a series of permanent magnets, and optically pumping the species between LFS and HFS states as they traverse the different magnetic field regions.^153^ After deceleration, these radicals can be confined in magnetic or magneto-optical traps (as described below), with the technique proposed to be applicable to other molecular radicals that can optically cycle at least a small number of photons.^152^

### 2.5 Reactions in traps

All of the preceding subsections have focused on reactions where the radical reactant is entrained within a beam: from crossed beam and flow-based methods to the use of merged and decelerated beams. While these techniques have seen widespread use in radical reaction dynamics studies, other approaches are starting to gain attention. In particular, there are many benefits associated with confining one or more of the reactants in a trap. Reactions occurring within a trap environment can be monitored for an extended period of time, enabling infrequent or slow processes to be examined, with a variety of sensitive detection methods available for probing the reaction products. Ion–radical reactions have been studied within traps for many years—with the ionic reactant spatially confined and the radical reactant subsequently introduced to the trap volume. Many of these ion–radical reaction systems have been studied within 22-pole traps, with reactions involving H atoms receiving particular attention. As this field has been recently (and thoroughly) reviewed, readers are directed to ref. 14, 33 and 154 for more details on ion–radical reaction dynamics.

**Fig. 4:**
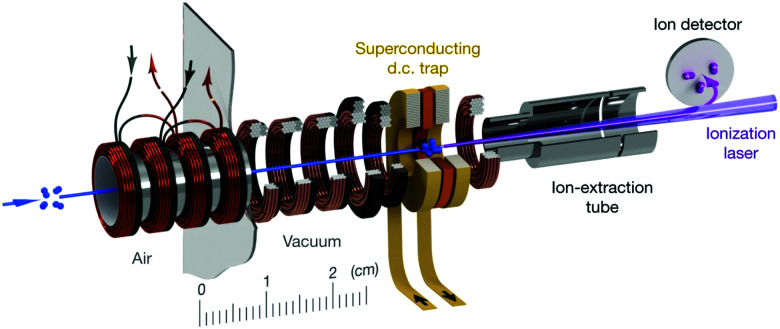
Schematic illustration of a travelling wave Zeeman decelerator and magnetic trap apparatus, for the co-deceleration and trapping of atomic and molecular radicals. Cold paramagnetic species enter the apparatus (as indicated by the blue arrow on the left) and are decelerated by a series of 480 co-moving magnetic traps (illustrated in bronze and black), formed by the application of current pulses to the coils. Slow-moving species at the end of the decelerator can be loaded into a superconducting trap. The trapped species are probed after a selected delay time using laser-based detection methods. Reproduced from Segev *et al.*, copyright 2019, under exclusive licence to Springer Nature Limited.^156^

Aside from ion–radical reaction studies within ion traps, there have only been a handful of radical reactions examined in other trap environments. This is primarily due to the challenges associated with traps for neutral species (such as radicals), which are typically much shallower than those for ionic species.^33,34^ Cryogenic environments and ultra-high vacuum conditions can extend the trap lifetime of radical species (up to 10s of seconds)—providing sufficient time for reactions to be examined.^155,156^ When reactions with trapped radicals do occur, only rarely can the neutral reaction products also be trapped (as they often do not possess the necessary properties to be confined by the fields, or may be formed with more kinetic energy than the depth of the trap). In spite of these challenges, a number of research groups have successfully combined Zeeman and Stark deceleration of radicals with magnetic and electrostatic trapping (as discussed in the preceding subsection), opening up the prospect of studying the reaction dynamics of cold, confined radical species such as OH, NH, O_2_, and CH_3_.^155–159^ Non-reactive collisions involving some of these species have already been observed—including, for example, a study of the ratio of elastic and inelastic O_2_–O_2_ and O_2_–Li collisions in a superconducting trap.^156^ Very recently, collisions between molecular oxygen and atomic carbon have been studied in a superconducting magnetic trap, with the reactants co-Zeeman decelerated prior to being loaded into the trap (see Fig. 4). A significant enhancement in the decay rate of trapped C(^3^P_1_) was observed when O_2_ was co-trapped, with the barrierless reaction C(^3^P_1_) + O_2_(^3^Σ^−^_g_) → CO(^1^Σ^+^) + O(^1^D) identified as a probable loss mechanism (in addition to elastic and inelastic collision-induced trap loss).^160^ These early results pave the way for detailed studies into the dynamics of the astrochemically important reaction between C and O_2_ at sub-Kelvin temperatures.

Buffer gas cooling methods have also been combined with magnetic and electrostatic trapping for molecular radicals including CaH and NH,^161,162^ with non-reactive NH + N collisions also examined within a magnetic trap.^162^ In the ultracold regime, impressive progress has seen the application of cooling and trapping techniques to a number of molecular radical species. For example, inelastic collisions between laser-cooled CaF molecules and Rb atoms, prepared in a magneto-optical trap (MOT) and subsequently confined in a magnetic quadrupole trap, have recently been studied at temperatures near 100 μK.^163^ Following the first report of ultracold Cs_2_ molecules in 2008,^164^ a handful of ultracold dimers have since been produced by the magnetoassociation of ultracold atoms—including radical dimers such as NaLi.^14,34,165^ While most studies involving ultracold atoms and molecules have focused on establishing elastic and inelastic scattering rates, there is certainly scope for the application of these methods to the study of reaction dynamics in the future—especially with the development of sensitive detection methods and the successful reaction dynamics study of the ultracold bimolecular reaction KRb + KRb.^166^

### 2.6 Alternative approaches

The preceding subsections identified a range of different methods that have been adopted for the study of radical reaction dynamics in the gas phase. Beyond these purely gas-phase studies, there are a number of alternative techniques that explore radical reactivity in related environments. For example, a range of radical reactions have been studied within superfluid helium nanodroplets. Droplets of ^4^He, typically composed of several thousand (or more) helium atoms, can reach temperatures as low as 0.37 K.^167^ Dopant species are incorporated into droplets as they pass through one or more ‘pick-up cells’ containing the reactant(s) of interest.^168^ Due to their superfluid nature, the helium droplets efficiently cool any captured dopant species, achieved through the evaporation of He atoms off the droplet surface. The inert nature and cold temperatures of He droplets make them an attractive medium for studying the reaction dynamics of reactive species such as radicals. The study of chemical dynamics within helium nanodroplets has been reviewed previously, with the reactions of several radical species explicitly discussed.^33,169–171^ As such, the following paragraph will mention only a few key benefits and applications of the technique. Readers are directed to previous work^33,169–171^ for a broader discussion on the topic.

While it is (usually) chemically inert, the helium environment can influence processes that occur within the droplet. For example, reaction intermediates can be stabilised by collisions with He atoms within the droplet. When collisional stabilisation occurs, it can reveal information about the underlying potential energy surface—such as confirming the presence of a submerged or energetic barrier along the reaction pathway. In certain systems, reactive intermediates can be directly probed within He droplets—something that is notoriously difficult to achieve in pure gas-phase measurements. The pre-reactive complex formed between OH and methanol was stabilised in a He nanodroplet, enabling spectroscopic measurements to be performed.^172^ The atmospherically important association reaction between OH and O_2_ has also been studied within He nanodroplets, with the formation of *trans*-HOOO found to proceed *via* a barrierless pathway.^173^ This finding was in contrast to expectations from theory work, where a significant energetic barrier was predicted to be present in the entrance channel.^174^ (Furthermore, the *cis*-HOOO isomer, anticipated from theory work to be near-isoenergetic with the *trans* species, was not detected experimentally.^173^) It took a further 7 years before multi-reference *ab initio* calculations could account for the experimental observations, with the “spurious” barrier along the reaction co-ordinate deemed to arise from non-adiabatic coupling to a nearby excited electronic state.^175^

Another field that is related to the gas-phase study of radical reaction dynamics is the probing of radical–surface interactions. A number of important processes occur at gas–surface interfaces, with the reactions of gas-phase radicals with aerosol surfaces (as occurs in the atmosphere) and radical reactions on the surface of interstellar dust particles particularly relevant to this highlight article.^176–186^ While the interactions of gas-phase radicals with surfaces are technically outside the scope of this work, they are mentioned here to highlight the benefits (to both fields) of combining methods for manipulating radical beams with techniques developed for the study of dynamics at surfaces. By exerting increased control over the radical reactant beam, it may be feasible to differentiate between competing reaction pathways and to elucidate reaction mechanisms in complex reaction systems. For example, plans are already underway to combine a magnetic radical filter with a liquid surface apparatus, for the study of radical–liquid surface collisions with state-selected and velocity-controlled radical species.^34,176,187^ This would represent a significant advancement beyond current capabilities and is an exciting future prospect.

## 3 Conclusions and future directions

In this highlight article, we have explored the reaction dynamics of paramagnetic species in the gas phase—focusing on recent experimental work conducted at low collision energies, where the properties of one (or both) reactants can be controlled. As is evident from the many reaction systems discussed, it is often challenging to predict the properties of radical reactions in the absence of detailed experiments and theory work. This complexity can arise from the very nature of the radical reactants (*i.e.*, the presence of one or more unpaired electrons), with more than one low-lying PES and non-adiabatic effects frequently needing to be considered. For example, despite the decades of attention given to ‘simple’ reactions such as F + H_2_, new dynamical features are still being uncovered.^47,48^ Even systems that were thought to be well understood can become more complex when a minor amendment is made. For example, integral cross sections recorded for the reaction between S + H_2_ were found to be well described by theory work, but features in the analogous S + HD reaction could not be immediately accounted for.^52,53^

Significant progress has been made in recent years, and a diverse range of experimental methods are now available for preparing radical reactants and sensitively detecting reaction products. A great degree of control can be exerted over the properties of paramagnetic species, often through the application of external magnetic fields. This ability to control and manipulate the properties of radicals has been critical in developing our understanding of low-energy reaction dynamics. For example, the use of magnetic guides in merged beam set-ups has seen radical reaction systems studied at collision energies below 10 mK, where quantum features come to the fore.^68^ When coupled with sensitive detection methods, such as coincidence measurements, exquisite detail about the reaction mechanism can be ascertained.^79^ In this way, low-energy and state-selected radical reaction studies have unveiled new insights into radical reactivity, with quantum features observed in a number of reaction systems. In other cases, stereodynamic effects have been successfully probed, with the importance of the spatial orientation of radical reactants explored. While this highlight article has intentionally focused on experimental studies, in many cases, state-of-the-art theory work can provide a more complete picture about how a given system reacts, extending the insights gained from experimental measurements. Studies that combine high-level experimental investigations with detailed theory work continue to represent the ‘gold standard’ in the study of reaction dynamics—with each component complementing (and sometimes challenging) the other.

The future prospects for the field are truly exciting. Work is underway to apply a number of techniques that have thus far been limited to a small subset of radical species to a larger range of reactants. For example, a number of research groups are actively working towards generating a range of ultracold open-shell molecules (such as CsYb, RbYb, RbSr and LiYb)^188^—with methods for studying ultracold bimolecular reaction dynamics recently developed.^166^ Zeeman decelerators and magnetic guides have been utilised for the precise study of inelastic scattering processes, with such methods also likely to be applicable to the study of reactive collisions.^61,67^ Impressive progress has also been seen in the co-deceleration and trapping of C with O_2_.^160^ These new combinations and applications of existing methods suggest that it will be possible to study more and more radical reaction systems in a controlled manner—at ultra-low collision energies, with full control over the reactant properties, and with the detection of product state distributions—in the coming years.

## Author contributions

All authors contributed equally to all aspects of the article.

## Conflicts of interest

There are no conflicts to declare.

## Supplementary Material
